# Characterization of New Oligosaccharides Obtained by An Enzymatic Cleavage of the Exopolysaccharide Produced by the Deep-Sea Bacterium *Alteromonas infernus* Using its Cell Extract

**DOI:** 10.3390/molecules24193441

**Published:** 2019-09-22

**Authors:** Katy Akoumany, Agata Zykwinska, Corinne Sinquin, Laëtitia Marchand, Mathieu Fanuel, David Ropartz, Hélène Rogniaux, Muriel Pipelier, Christine Delbarre-Ladrat, Sylvia Colliec-Jouault

**Affiliations:** 1 Ifremer, Laboratoire Ecosystèmes Microbiens et Molécules Marines pour les Biotechnologies, F-44311 Nantes, France; Katy.Akoumany@ifremer.fr (K.A.); Corinne.Sinquin@ifremer.fr (C.S.); Laetitia.Marchand@ifremer.fr (L.M.); Christine.Delbarre.Ladrat@ifremer.fr (C.D.-L.); Sylvia.Colliec.Jouault@ifremer.fr (S.C.-J.); 2 Université de Nantes, CNRS, Chimie et Interdisciplinarité: Synthèse, Analyse, Modélisation (CEISAM), UMR CNRS 6230, Faculté des Sciences et des Techniques, F-44322 Nantes, France; Muriel.Pipelier@univ-nantes.fr; 3 INRA, UR1268 Biopolymères Interactions Assemblages, F-44300 Nantes, France; Mathieu.Fanuel@inra.fr (M.F.); David.Ropartz@inra.fr (D.R.); Helene.Rogniaux@inra.fr (H.R.)

**Keywords:** deep-sea bacterium, *Alteromonas infernus*, wild-type strain, exopolysaccharides, glycosaminoglycan-mimetic, enzymatic depolymerization, structural analysis, mass spectrometry

## Abstract

Bacteria from deep-sea hydrothermal vents constitute an attractive source of bioactive molecules. In particular, exopolysaccharides (EPS) produced by these bacteria become a renewable source of both biocompatible and biodegradable molecules. The low molecular weight (LMW) derivatives of the GY785 EPS produced by the deep-sea hydrothermal vent strain *Alteromonas infernus* have previously displayed some biological properties, similar to those of glycosaminoglycans (GAG), explored in cancer and tissue engineering. These GAG-mimetic derivatives are obtained through a free radical depolymerization process, which could, however, affect their structural integrity. In a previous study, we have shown that *A. infernus* produces depolymerizing enzymes active on its own EPS. In the present study, an enzymatic reaction was optimized to generate LMW derivatives of the GY785 EPS, which could advantageously replace the present bioactive derivatives obtained by a chemical process. Analysis by mass spectrometry of the oligosaccharide fractions released after enzymatic treatment revealed that mainly a lyase activity was responsible for the polysaccharide depolymerization. The repeating unit of the GY785 EPS produced by enzyme cleavage was then fully characterized.

## 1. Introduction

Polysaccharides are macromolecules present ubiquitously in plant (cellulose, pectin) [1], seaweed (alginate, carrageenan) [2,3], and animal (hyaluronan, chitin) [4] tissues. They can also be produced by microorganisms, such as bacteria (dextran, gellan, xanthan, hyaluronic acid) [5]. Polysaccharides are characterized by a repeating unit composed of osidic residues linked by glycosidic bonds, either in linear or branched structures. Monosaccharides may differ in nature, as acidic, neutral and amino sugars can be distinguished. These residues may be substituted by organic and/or inorganic substituents, such as acetate, succinic, pyruvic acid, sulfate and phosphate groups [6]. All these structural features are crucial for the functional, i.e., physico-chemical and biological properties of exopolysaccharides (EPS). Indeed, due to their gelling, thickening and stabilizing properties, polysaccharides, mainly pectin, alginate, gellan, xanthan and hyaluronic acid are widely used in food, cosmetic and pharmaceutical industries [7,8]. Polysaccharides from animal tissues belonging to glycosaminoglycan (GAG) family (heparin, heparan sulfate, chondroitin sulfate, dermatan sulfate, keratan sulfate and hyaluronic acid) are now well recognized for their ability to regulate many biological processes. They are localized on the cell surface and in the extracellular matrix (ECM) of connective tissues, where they are involved in cell-cell and cell-matrix interactions [9]. Indeed, GAG are able to interact with several proteins, such as cytokines, chemokines, growth factors and enzymes. Non-covalent interactions, such as ionic interactions, hydrogen bonding, hydrophobic interactions and van der Waals forces are mainly responsible for the protein binding [10]. Moreover, GAG has a specific structure. They are all linear anionic heteropolysaccharides with molar masses ranging from 10,000 to 100,000 g/mol, except for hyaluronic acid characterized by its high molecular weight (HMW) [10]. Their repeating unit is composed of a disaccharide constituted of amino sugar and uronic acid (or neutral sugar), which are both frequently *N-* and/or *O*-sulfated, excepting hyaluronic acid devoid of sulfates. However, the main GAG disadvantage for their biomedical applications is their structural heterogeneity, which implies the implementation of complex extraction and purification steps for their isolation from animal tissues [11,12]. To avoid these drawbacks, animal-free GAG-mimetics are under intense development. Synthesis of well-defined GAG oligosaccharide sequences by organic chemistry constitutes an alternative solution [13]. These derivatives can be obtained by hemi-synthesis, starting from isolated GAG fragments, or by total synthesis, generally using monosaccharide building blocks. Usually, the minimal sequence required for the recognition process was composed of 5–6 monosaccharides, which implied a long multi-step organic synthesis. The best example is the first total synthesis of the heparin pentasaccharide sequence having a high affinity for antithrombin [14,15]. After optimization, this synthesis led to Fondaparinux drug used for the prevention of venous thromboembolic events (Arixtra^®^, Sanofi-Synthélabo/Organon) [16]. A chemo-enzymatic approach constitutes another strategy to produce GAG-like oligosaccharides. Indeed, this approach allows a synthesis under mild conditions, with a high glycosidic bond stereoselectivity and sulfation regioselectivity without the necessity for hydroxyl protection/deprotection strategy. Hydrolases and transferases are generally the two classes of enzymes typically used [17]. Recently, Xu et al. [18] have developed the chemo-enzymatic synthesis of two dodecasaccharides started from the commercially available acceptor *p*-nitrophenyl glucuronide detectable in UV and the uridine diphosphate (UDP)-sugar as a donor [18]. A glycosyltransferase, an epimerase and several sulfotransferases were used to obtain their expected oligosaccharides in multigram-scale after ~20 steps. The chemo-enzymatic synthesis of 66 well-defined heparan sulfate and heparin oligosaccharides with another UV detectable glycosyl acceptor, *N*-(6-azidohexanamidyl) *p*-aminophenylglucuronide has also been reported [19].

Bacteria may also constitute a source of animal-free GAG. *Pasteurella multocida* produces a capsular polysaccharide with the chemical structure of HMW heparosan (~300,000 g/mol), a precursor in the biosynthesis of heparin and heparan sulfate, while *Escherichia coli* K5 synthesizes low molecular weight (LMW) derivatives, with a molecular weight ranging from 10,000 g/mol to 20,000 g/mol [20,21]. Another non-sulfated polysaccharide produced by the strain *E. coli* K4 had a structure similar to non-sulfated chondroitin sulfate [22]. The production of bacterial hyaluronic acid is now ensured by *Streptococcus zooepidemicus,* in order to avoid its extraction from mammalian tissues. Different culture conditions were described to improve its production for future applications for cosmetic and biomedical applications [23,24].

**Figure 1:**
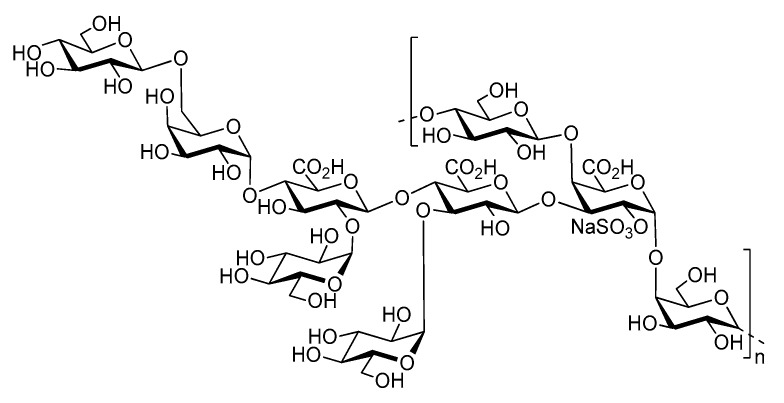
Structure of the GY785 exopolysaccharide (EPS) repeating unit [27].

Marine bacteria have also been shown to produce exopolysaccharides (EPS) [25]. In particular, deep-sea hydrothermal vent bacteria have been identified as a rich source of EPS of unusual chemical compositions and structures [6,26]. Among these bacteria, the mesophilic, aerobic and Gram-negative bacterium *Alteromonas infernus* produces HMW (2,000,000 g/mol) slightly sulfated (3 wt%S) GY785 EPS. The nonasaccharidic repeat unit of the native EPS is represented in Figure 1 [27]. Its main chain consists of a trisaccharide, where the central galacturonic acid (GalA) is substituted by glucose (Glc) and galactose (Gal) residues. GalA residue of the main chain is sulfated at *O*-2 and substituted at *O*-3 by a side chain composed of two glucuronic acids (GlcA), one Glc and one Gal in the sequence: β-d-Glc*p*-(1→6)-α-d-Galp-(1→4)-β-d-GlcpA-(1→4)-β-d-GlcpA-(1→. Each GlcA is substituted by a terminal Glc.

The biological activities of both native EPS and oversulfated LMW EPS derivatives, prepared by free radical depolymerization and chemical sulfation, have been studied. For anti-cancer activity, the oversulfated (30% of sulfate) LMW EPS derivative (15,000 g/mol) was evaluated in vitro and in vivo against osteosarcoma [28]. These derivatives decreased both the migration and invasiveness of osteosarcoma cells in vitro and inhibited lung metastases in vivo. In cartilage tissue engineering, LMW EPS derivatives (15,000 g/mol) and their oversulfated counterparts (20,000 g/mol) favored the chondrogenic differentiation of mesenchymal stem cells in vitro [29].

Although the chemical process used to obtain LMW EPS derivatives improves their biological activities, the structural integrity of these derivatives may not be conserved, and various polysaccharidic chains with structural heterogeneity could be generated. The formation of anhydrosugar after chemical depolymerization was reported [30,31]. An alternative approach to produce more homogeneous LMW derivatives with a milder process could be an improvement, such as enzymatic depolymerization, which enables to preserve the structural integrity due to the specificity of glycosidic bond cleavage. According to the carbohydrate active enzymes (CAZy) classification, three of the five catalytic families can be involved in the glycosidic bond cleavage: Glycoside Hydrolases (GHs) by hydrolysis, Polysaccharide Lyases (PLs) by a *β*-elimination mechanism and Auxiliary Activities (AAs) by oxidation [32,33]. Recently, it was reported that *A. infernus* produces endogenous enzymes able to depolymerize its own EPS, i.e., GY785 EPS [34]. Indeed, after cell lysis, depolymerizing activities were observed in both soluble lysate and insoluble cell debris. The structural analysis of small oligosaccharides (<1500 g/mol), released after incubation of the EPS with a soluble lysate (L), evidenced several depolymerizing activities, amongst lyase, hydrolase and sulfatase activities [34].

The aim of the present study was to go further into the enzymatic depolymerization process and optimize the reaction to generate LMW EPS derivatives that could advantageously replace the bioactive derivatives usually obtained through the chemical approach.

## 2. Results and Discussion

### 2.1. Evaluation of the Depolymerization by Gel Electrophoresis and High Pressure Size Exclusion Chromatography with Multi Angle Light Scattering (HPSEC-MALS)

**Figure 2:**
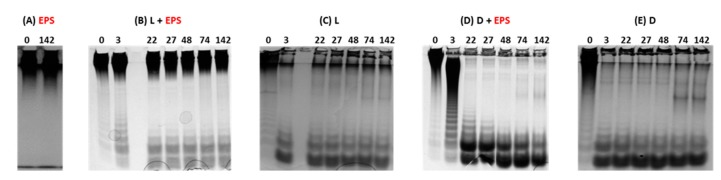
PAGE analysis of the native HMW GY785 EPS, EPS (**A**), EPS incubated with soluble cell lysate, L + EPS (**B**), soluble cell lysate, L (**C**), EPS incubated with insoluble cell debris, D + EPS (**D**), insoluble cell debris, D (**E**) in Tris HCl buffer at different incubation times (0, 3, 22, 27, 48, 74 and 142 h).

To generate LMW EPS derivatives that are usually prepared through free radical depolymerization process, the enzymatic method was applied to the native HMW GY785 EPS (2,000,000 g/mol). After bacterial cell lysis, performed in the presence of Tween 20 surfactant, two protein extracts were obtained: soluble lysate (L) and insoluble cell debris (D). Protein extracts (L or D) were incubated with the native EPS, and the depolymerization was followed at different incubation times for 142 h. To rapidly assess the EPS degradation, recovered samples were firstly analyzed on polyacrylamide gel electrophoresis (PAGE). To reveal the presence of anionic polysaccharide fragments, Stains All cationic dye was used. No migration pattern was observed for the native HMW EPS (Figure 2A). Indeed, a large band was present on the top of the gel during the whole incubation period of 142 h. When the native EPS was incubated with the lysate, L + EPS, several bands of low intensity were observed at the bottom of the gel, starting as early as 3 h of incubation (Figure 2B). However, these bands were also observed for the lysate alone and could result from the degradation of cellular components present in the soluble lysate (Figure 2C). In addition, a band, characteristic of the HMW EPS, was still visible on the top of the gel. All these observations suggest that only low depolymerization was induced by the lysate. In contrast, after only 3 h of incubation with the insoluble cell debris, D + EPS (Figure 2D), a broad smear on the top of the gel, as well as intense bands at the bottom of the gel were observed. Over 3 h of incubation, the extended smear observed at 3 h disappeared, and bands with a high intensity were clearly visible at the bottom of the gel. Since no band corresponding to the native EPS was observed on the top of the gel, these strong bands resulted mainly from the EPS depolymerization. Their intensity was, however, enhanced by the degradation of some cellular components present in the cell debris (Figure 2E).

However, this is not clear why the higher depolymerizing activity was observed in the insoluble cell debris (D), compared to the soluble cell lysate (L), when Tween 20 surfactant was used. The addition of surfactant did not enhance enzyme solubilization and their recovery in the soluble lysate. However, the presence of surfactant could improve the EPS accessibility to membrane enzymes and/or stabilize the EPS into the conformation that favors its depolymerization, through weak non-covalent interactions, such as hydrogen bonding and van der Waals forces between the polysaccharide backbone and the surfactant molecules.

**Figure 3:**
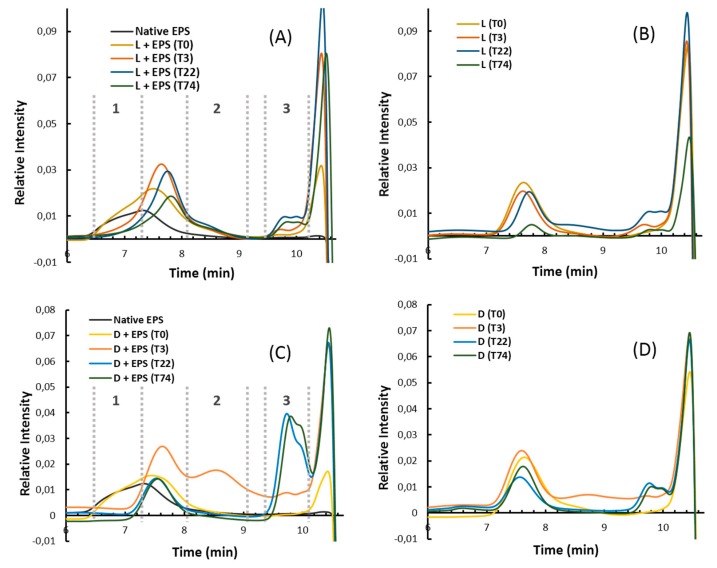
High pressure size exclusion chromatography (HPSEC) profiles (refractive index (RI) detection) of the native GY785 EPS incubated with lysate, L + EPS (**A**), lysate, L (**B**), EPS incubated with insoluble cell debris, D + EPS (**C**) and cell debris, D (**D**) in Tris HCl buffer for different incubation times (T) 0, 3, 22 and 74 h.

In order to confirm enzymatic depolymerizing activities of the protein extracts, L and D, samples were analyzed by high pressure size exclusion chromatography (HPSEC) and the elution profiles are presented in Figure 3. When the native HMW EPS was incubated with the lysate, a large peak eluted between 6.5 and 8 min was observed at the beginning of the incubation with enzymes (0 h) (Figure 3A). This peak overlays the peaks that correspond to the native EPS and the lysate alone (Figure 3A,B). Indeed, the peak corresponding to the lysate was eluted between 7 and 8 min and may arise from the presence of nucleic acids, proteins and other macromolecules (e.g., peptidoglycans) present in bacterial cells, which were co-extracted during the sample preparation by cell lysis. Degradation of some cellular components led to a decrease in the peak at 7.5 min and an increase in the peak at 9.5 min (Figure 3B), in agreement with PAGE analysis. The intense peak after 10 min corresponded to salts and very low molecular weight components eluted at the total volume of the column. After 3 h of incubation, a significant shift of eluted masses was observed, with the partial loss of the HMW EPS population at 7 min elution (zone 1) and an occurrence of LMW populations at 10 min elution (zone 3). Increasing incubation time (from 22 h to 74 h) led to only a slight increase in LMW EPS population intensity. However, the incubation of the native EPS with cell debris led to rapid depolymerization within the first 3 h (Figure 3C). Indeed, HMW EPS population completely disappeared (zone 1), and new populations were clearly observed in zones 2 and 3. The population of intermediate molecular weights eluted between 8 and 9 min (zone 2) was not generated when the EPS was incubated with the lysate (Figure 3A). Further incubation with the cell debris induced a total loss of the population of intermediate molecular weights presented in zone 2 along with the generation of an LMW EPS population eluted between 9.5 and 10 min (zone 3). By taking into account that the peak eluted between 7 and 8 min corresponds to cell debris (Figure 3C,D), rich in nucleic acids, proteins and other macromolecules present in bacterial cells, it can be concluded that over 22 h, the native HMW EPS was completely depolymerized. HPSEC results were, therefore, in agreement with PAGE observations (Figure 2).

**Figure 4:**
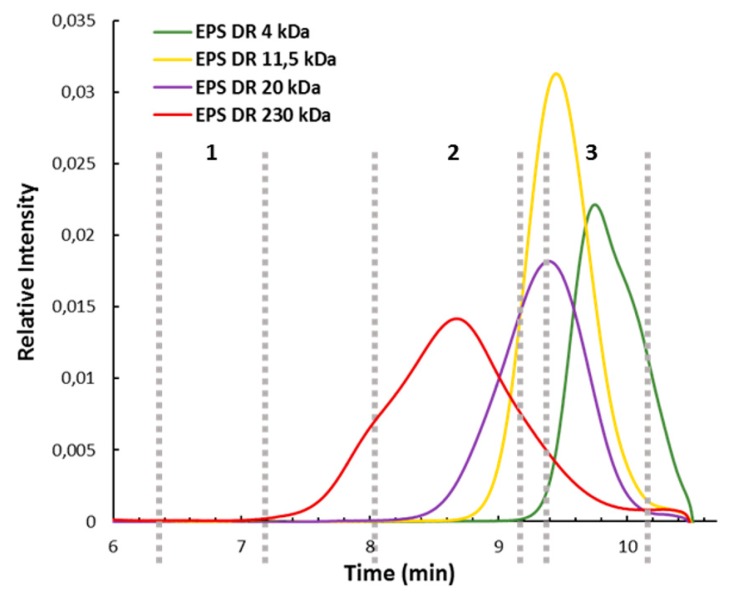
High pressure size exclusion chromatography with multi angle light scattering (HPSEC-MALS) profiles of the GY785 EPS derivatives (EPS DR) of weight-average molecular weights of 4000, 11,500, 20,000 and 230,000 g/mol.

High pressure size exclusion chromatography with multi angle light scattering (HPSEC-MALS) elution profiles of four GY785 EPS derivatives with different molecular weights ranging from 4000 to 230,000 g/mol, obtained by free radical depolymerization (Figure 4), indicated that the derivatives generated after 3 h of incubation of the native GY785 EPS with cell debris presented a weight-average molecular weight close to 200,000 g/mol (Figure 3C, zone 2). After 22 h of incubation, LMW derivatives of weight-average molecular weights ranging from 4000 to 15,000 g/mol were mainly produced (Figure 3C, zone 3).

### 2.2. Characterization of Oligosaccharide Fractions Generated by Insoluble Cell Debris

**Figure 5:**
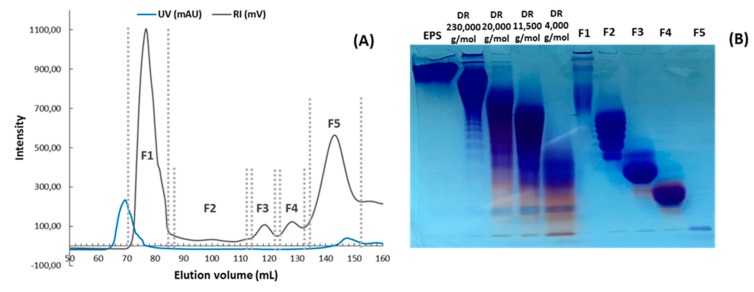
SEC fractionation of the native GY785 EPS depolymerized with cell debris, D for 63 h; UV and RI detections are indicated in blue and grey, respectively (**A**). PAGE analysis of the native GY785 EPS, four EPS derivatives with the weight-average molecular weight of 4000, 11,500, 20,000 and 230,000 g/mol, respectively, and five SEC fractions obtained after the GY785 EPS incubated with cell debris (**B**).

In order to purify and characterize the LMW EPS derivatives of weight-average molecular weights ranging from 4000 to 20,000 g/mol (Figure 3C, zone 3), the native GY785 EPS, depolymerized with cell debris for 63 h, was fractionated by size-exclusion chromatography (SEC). A Sephacryl^®^ S100HR column, allowing a fractionation ranging from 1000 to 100,000 g/mol, was used. After the fractionation step, five main fractions were isolated (Figure 5A). Fraction F1 was recovered with the highest amount (34.6 mg), whereas fraction F5 gave only 0.5 mg of dry matter (Table 1). By taking into account the fact that 50 mg of the EPS were depolymerized and that 40.4 mg was recovered for five fractions, probably smaller oligosaccharides were also obtained and eluted with salts. However, less depolymerized EPS fractions of higher molecular weight could also be present and eluted with other residual components present in cell debris, such as nucleic acids, proteins and lipopolysaccharides within the fraction F1, as revealed by UV detection in Figure 5A. The five fractions were analyzed by PAGE together with the GY785 DR standards obtained by free radical depolymerization (Figure 5B). Fraction F1 showed the presence of polysaccharide derivatives of intermediate molecular weight (230,000 g/mol), while fractions F2 to F4 corresponded to oligosaccharides with a molecular weight ranging from 4000 (F4) to 20,000 (F2) g/mol. Fraction F2 appeared, however, highly heterogeneous as several bands were clearly visible. Fraction F5 at the bottom of the gel presented a thin band with very low intensity in accordance with the very low amount of product recovered (Table 1). The low amount recovered for fraction F5 (0.5 mg), although the corresponding chromatographic peak displayed high intensity, can be explained by the additional presence of salts (ammonium bicarbonate from the buffer) eluted at the total volume of the column and detected by RI. However, since the pooled fractions were freeze-dried, salts from the buffer were evaporated.

The osidic composition was determined for each fraction except for F5 (recovered in too low amount) and compared to the composition of the native GY785 EPS (Table 2). The main characteristic osidic residues of the native polysaccharide (Gal, Glc and GlcA) were detected in all four fractions; however, their molar ratios were lower than in the native EPS. GalA residue was present only in F3 and F4. Besides the typical residues constitutive of the native polysaccharide, other sugars, i.e., Rha, Fuc and Man were also present in F1. To assess if these osidic residues originate from cell debris (D), sugar analysis of this protein extract was also performed. Rha, Fuc and Glc were mainly detected, suggesting that these residues could originate from cell debris, a complex mixture of various cell membrane molecules. Rha and Fuc residues may indicate the presence of methyl-pentose rich oligosaccharides, which can be associated with cell membranes, and are, thus, recovered during cell lysis, as previously suggested [34].

### 2.3. Oligosaccharide Structure Characterization by Mass Spectrometry

Mass spectrometry (MS) experiments were performed in order to elucidate the structure of the oligosaccharides released by enzymes present in the insoluble cell debris extract. To screen the species, fractions F2, F3, F4 and F5, which showed the smallest species according to PAGE analysis (Figure 5B), were characterized by Ion-Pairing Reverse-Phase Ultra-High Performance Liquid Chromatography coupled to mass spectrometer (IP-RP)-UHPLC-MS. Structures presented in Table 3 were hypothesized according to the GY785 EPS repeating unit previously described (Figure 1, [27]), as well as oligosaccharides released by enzymes described in our previous work [34]. According to the exact mass measurement and the retention time in (IP-RP)-UHPLC-MS, which is correlated with the number of sulfate group of the oligosaccharide, a doubly sulfated octasaccharide was identified in fraction F5 (Table 3, structure A). This octasaccharide contains an unsaturation in its structure, indicating a lyase activity, which introduces a double bond to the non-reducing moiety of the released product. Since a lyase was involved in glycosidic bond cleavage between Glc and GalA residues of the main chain, this activity remains responsible for the main chain depolymerization. The octasaccharide structure differs slightly from the GY785 EPS repeating unit [27]. Indeed, one hexose residue (Glc), usually linked to the Gal residue, of the side chain [27,34] was replaced by a sulfate group. Fractions F4, F3 and F2 contain respectively a dimer, a trimer and a mixture of a tetra-, penta- and hexamer of the oligosaccharide described in F5. In addition, in the four fractions, species with a lower number of hexoses (indicated in Table 3 as supplemental species) were also identified, which can indicate some additional hydrolase activities in the insoluble cell debris extract, as previously observed [34].

**Figure 6:**
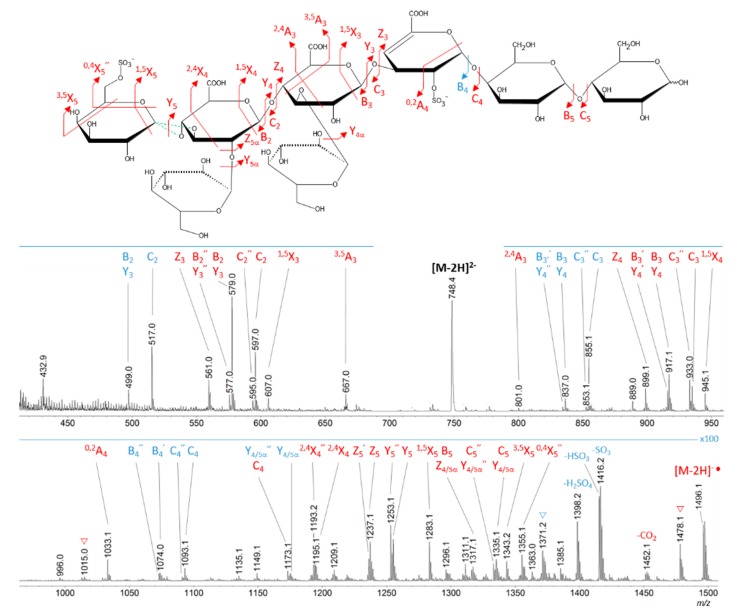
Helium charge transfer dissociation (He-CTD) tandem MS spectrum of the structure A isolated as a [M − 2H]^2−^ species at *m*/*z* 748.4 in negative ionization mode. For better readability, the mass range was split into two parts. Red, fully sulfated fragments; Blue, fragments with one sulfate loss; Triangle, water losses.

In order to confirm the structures detected in (IP-RP)-UHPLC-MS, the structure A observed in F5 (Table 3), which represents the “building block” of all the detected species, was further studied using helium charge transfer dissociation (He-CTD) tandem mass spectrometry (MS/MS) [36] in negative ionization mode [37]. The complete structure of the octasaccharide presented on the He-CTD MS/MS spectrum (Figure 6) was unequivocally characterized, except for the linkage (1,3 or 1,4) between the sulfated Gal and the GlcA at the end of the side chain. MS/MS information indicated that i) a lyase activity is involved in the EPS depolymerization because of the double bond on the GalA residue of the main chain (indicated by the C_4_, ^0,2^A_4_, Z_3_ and Y_3_) and ii) a sulfate group replaces the Glc residue on the C6 position of the Gal residue of the side chain (indicated by the intracyclic fragments ^0,4^X_5_”, ^3,5^X_5_ and ^1,5^X_5_ according to the nomenclature of Domon and Costello [38]). It can be thought that the doubly sulfated octasaccharide identified for the first time in this study co-exists with the nonasaccharide containing one sulfate group initially described for the GY785 EPS repeating unit [27]. During EPS biosynthesis, the side chain may be substituted by either a terminal Glc residue or a terminal sulfate group, while the core of the repeating unit stays intact. For example, in the case of GAG biosynthesis, differences in sulfation pattern are determined by the activity of the enzymes, availability of precursors and intracellular flux or traffic during the biosynthesis process [39].

## 3. Conclusions

In order to produce bioactive LMW EPS derivatives from the native HMW GY785 EPS, an enzymatic approach was applied. Until now, LWM EPS derivatives were prepared by a chemical process, which remains, however, nonspecific and leads to derivatives with a structural heterogeneity. Previously, protein extracts prepared from *A. infernus* cells have been shown to contain endogenous depolymerizing enzymes, i.e., a lyase and hydrolases, active on the GY785 EPS produced by the bacterium. In this study, the enzymatic approach applied to the native EPS led to LMW EPS derivatives of intermediate molecular weight ranging from 4000 to 230,000 g/mol. Mass spectrometry performed on oligosaccharide fractions recovered after a preparative size-exclusion chromatography allowed to confirm the depolymerizing activities previously observed, with a lyase activity responsible for the main chain cleavage. The smallest oligosaccharide recovered within the fractions was composed of eight osidic residues and corresponded to the GY785 EPS repeating unit previously described, except for the terminal Glc residue of the side chain substituted by a sulfate group. This modification appears most likely during EPS biosynthesis, leading to two types of repeating unit constituted of an octasaccharide with two sulfate groups and a nonasaccharide having only one sulfate.

## 4. Materials and Methods

### 4.1. Production of the Native GY785 EPS

Production of the GY785 EPS by *A. infernus* and its purification were previously described [40]. Here, GY785 EPS production was performed at 25 °C pH 7.4 in a 30 L fermenter (Techfors 30 L INFORS, Switzerland). Then, 20 L of Zobell medium, containing 5 g/L of tryptone, 1 g/L of yeast extract, and 33.3 g/L of aquarium salts were introduced in the fermenter, and 2 L of cell suspension inoculum was then added. For EPS biosynthesis, 30 g/L of glucose was added at the beginning of the batch. At the end of the fermentation process (48 h), the EPS excreted in its soluble form and remaining in the culture medium was separated from bacterial cells by a centrifugation step. The supernatant was then ultrafiltrated on a 100 kDa cut-off membrane and freeze-dried.

### 4.2. Enzymatic Assays Using A. infernus Protein Extracts

For *A. infernus* protein extract preparation, 500 mL of Zobell medium, two-times concentrated, containing 8 g/L of tryptone, 2 g/L of yeast extract and 66.6 g/L of aquarium salts, and 500 mL of glucose solution at 60 g/L was prepared. All solutions were autoclaved. Then, 20 mL of cell suspension inoculum was added to the medium, and the cells were incubated for 48 h at 30 °C under agitation (175 rpm). After centrifugation at 10,000 g for 40 min at 10 °C, the supernatant was not conserved. To preserve bacterial cell integrity, the pellet was suspended in 10 mM Tris HCl buffer at pH 8 containing 20 g/L NaCl and was centrifuged at 10,000 g for 30 min at 10 °C. The bacterial pellet was then suspended in 80 mL of 50 mM Tris HCl buffer at pH 8 containing protease inhibitors (cOmplete Tablets EDTA-free, Roche, Basel, Switzerland) and 1% Tween 20 (Merck, Darmstadt, Germany). Cell lysis was performed by sonication of 10 mL aliquots during seven cycles of 1 min with 1 min pause. Soluble lysate (L) was isolated from the insoluble cell debris (D) after centrifugation at 10,000 g for 20 min at 10 °C.

Enzymatic depolymerization assays were performed by incubating one volume of the GY785 EPS solution at 4 mg/mL in 50 mM Tris HCl buffer pH 8 containing 0.02% NaN_3_ with one volume of the cellular extract (L or D) at 30 °C under agitation (175 rpm). Samples were removed at 0, 3, 22, 27, 48, 74 and 142 h, heated at 100 °C during 7 min, centrifuged at 10,000 g for 15 min at room temperature and the supernatants were kept at −20 °C for further analysis.

### 4.3. Batch Depolymerization Using A. infernus Cell Debris

To produce oligosaccharides, 15 mL of the GY785 EPS solution at 4 mg/mL in 50 mM Tris HCl buffer pH 8 containing 0.02% NaN_3_ was incubated with 15 mL of cell debris (D) under agitation for 63 h at 30 °C (175 rpm). After 63h, the final solution was heated at 100 °C for 7 min, centrifuged at 10,000 g for 15 min at room temperature. Then, 25 mL of the supernatant was fractionated on a Sephacryl^®^ S-100-HR column (200 mL) at a flow rate of 1 mL/min with 0.1 M ammonium bicarbonate as eluent buffer. Fractions were pooled before being freeze-dried and conserved at room temperature until analysis.

### 4.4. Electrophoresis in Polyacrylamide Gels (PAGE)

20% polyacrylamide separating gel was prepared in 1.5 M Tris HCl buffer at pH 8.8 containing ammonium persulfate (0.05% *w*/*v*) and tetramethylethylenediamine (TEMED). Five percent polyacrylamide stacking gel was prepared in 0.5 M Tris HCl at pH 6.8, ammonium persulfate (0.05% *w*/*v*) and TEMED. Then, 20 μL of samples prepared in loading buffer (0.5 M Tris HCl pH 6.8, glycerol, 0.5 M EDTA, 0.5% *w/v* bromophenol) were then loaded on polymerized acrylamide gels. Gels were fixed for 4 h in 25% (*v*/*v*) isopropanol and then colored overnight in the dark by Stains All (3,3′-Diethyl-9-methyl-4,5,4′,5′-dibenzothiacarbocyanine) solution at 0.005% prepared, as follows: Five milliliters of a mother 0.1% Stains All solution in dimethylformamide (*w*/*v*); 5 mL of 300mM Tris–HCl pH 8.8; 5 mL of dimethylformamide; 25 mL of isopropanol; 60 mL of H_2_O [41]. They were then destained for 5 h under natural light.

### 4.5. HPSEC-MALS

High Performance Size-Exclusion Chromatography (HPSEC) coupled with a multiangle light scattering (MALS, Dawn Heleos-II, Wyatt Technology, Santa Barbara, CA, USA) and a differential refractive index (RI) (Optilab Wyatt technology, Santa Barbara, CA, USA) detectors allow to follow the depolymerization. HPSEC system was composed of an HPLC system Prominence (Shimadzu Co, Kyoto, Japan), a PL aquagel-OH mixed, 8 μm (Agilent Technologies, Santa Clara, CA, USA) guard column (U 7.5 mm × L 50 mm), and a PL aquagel-OH mixed (Agilent Technologies, Santa Clara, CA, USA) separation column (U 7.5 mm × L 300 mm). The eluent was 0.1 M ammonium acetate. The molecular weight was calculated using a refractive index increment characteristic of polysaccharides, dn/dc = 0.145 mL/g.

### 4.6. Monosaccharide Composition

Monosaccharide composition was determined, according to Kamerling et al. [42] method, modified by Montreuil et al. [43]. Samples were hydrolyzed for 4 h at 100 °C by 3M MeOH/HCl with myo-inositol used as the internal standard. After re-N-acetylation with acetic anhydride overnight at room temperature, the methyl glycosides were converted into their corresponding trimethylsilyl derivatives using *N*,*O*-bis(trimethylsilyl)trifluoroacetamide and trimethylchlorosilane (BSTFA:TMCS) 99:1 (Merck, Darmstadt, Germany). Separation and quantification of the per-*O*-trimethylsilyl methyl glycosides were performed by gas chromatography (GC-FID, Agilent Technologies 6890N).

### 4.7. Protein Content

The protein content in the EPS samples was determined using bicinchoninic acid method (BCA-Kit, Sigma, St. Louis, MO, USA).

### 4.8. UHPLC-MS

Ion pairing-reversed phase chromatography separations were run on an ultra high performance liquid chromatography system (UHPLC, Acquity H-Class^®^ Waters, Manchester, UK), equipped with a BEH C18 column (100 mm × 2.1 mm, packed with 1.7 μm porosity particles) (Waters, Manchester, UK). The flow rate was of 0.150 mL min^−1^ and column was heated at 30 °C. A ternary gradient was used (A, pure water; B, pure acetonitrile; and C, 20 mM hexylammonium dissolved in water, and pH value adjusted to 6 by addition of acetic acid), from 16.6% to 35% of solvent B in 10 min, then up to 63.4% at 20 min and maintained at 73.4% for 4.5 min. Percentage of solvent C was kept constant at 25%.

MS measurements were done through a direct coupling with a Synapt G2Si high-definition mass spectrometer (Waters Corp., Manchester, UK) on a mass range of 300–2000 *m*/*z*. The instrument was operated in a negative ionization mode in the so-called sensitivity mode, with an ESI capillary voltage of 2.5 kV and a sampling cone voltage of 50 V. Data acquisition was carried out using MassLynx software (V4.1).

### 4.9. He-CTD-MS/MS

Fraction F5 was analyzed with the use of a modified AmaZon 3D ion trap (Bruker Daltonics, Billerica, MA, USA) as described in [37]. The sample was infused at a flow rate of 5 μL/min. The mass spectrometer operated in negative polarity, with an ESI capillary voltage of 3.5 kV. Raw data were processed with mMass 5.3.0 [44].

## Figures and Tables

**Table 1:** Amount (mg) of fractions recovered after size exclusion chromatography.

Fraction	F1	F2	F3	F4	F5
**Amount (mg)**	34.6	2.4	1.4	1.5	0.5

**Table 2:** Osidic composition (molar ratio) of the native GY785 EPS and the four fractions, recovered after SEC fractionation, and insoluble cell debris (D).

	Osidic Composition (Molar Ratio)						
	Rha	Fuc	Man	Gal	Glc	GalA	GlcA
**GY785 EPS**	0.2	0.1	0.4	3.6	4.7	1.0	2.0
**F1**	1.0	0.4	0.5	0.3	1.0	0	1.6
**F2**	0	0	0	0.1	1.1	0	1.0
**F3**	0	0	0	1.0	1.6	0.2	1.0
**F4**	0	0	0	0.7	1.1	0.1	1.0
**Cell debris (D)**	0.3	0.1	0	0	1.0	0	0

Rha, rhamnose; Fuc, fucose; Man, mannose; Gal, galactose; Glc, glucose; GalA, galacturonic acid; GlcA, glucuronic acid.

**Table 3:** UHPLC-MS analysis of the fractions obtained after SEC fractionation of the native GY785 EPS depolymerized with cell debris extract. Structures are schematized according to the Symbol Nomenclature for Glycans (SNFG) [35]. 4//, unsaturation between C4 and C5 of GalA of the main chain. IPR, ion pair reagent; Supp. species, supplemental species.

		Structure	Monoisotopic Mass (Theoretical, Da)	RetentionTime (min)	Measured *m*/*z*	Adduct
**F5**	A	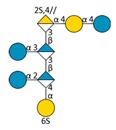	1498.27	2.73	748.13	[M − 2H]^2−^
	**Supp. Species: A—(1** **⇨** **3 hexoses)**		1336.22 ⇨ 1012.12	2.76 ⇨ 2.88	667.10 ⇨ 505.05	[M − 2H]^2−^
**F4**	B	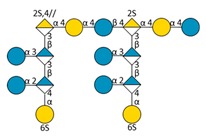	2996.55	7.00	1031.55	[M − 4H + 1IPR]^3−^
	**Supp. Species: B—(1** **⇨** **4 hexoses)**		2834.5 ⇨ 2348.34	7.05 ⇨ 7.21	977.52 ⇨ 815.47	[M − 4H + 1IPR]^3−^
**F3**	C	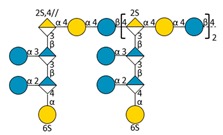	4494.82	8.56	1173.26	[M − 6H + 2IPR]^4−^
	**Supp. Species: C—(1** **⇨** **5 hexoses)**		4332.77 ⇨ 3684.56	8.59 ⇨ 8.84	1132.75 ⇨ 970.69	[M − 6H + 2IPR]^4−^
**F2**	D	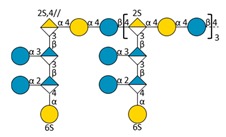	5993.10	9.69	1598.38	[M − 8H + 4IPR]^4−^
	**Supp. Species: D—(1** **⇨** **5 hexoses)**		5831.04 ⇨ 5182.83	9.69 ⇨ 9.79	1557.88 ⇨ 1395.82	[M − 8H + 4IPR]^4-^
	E	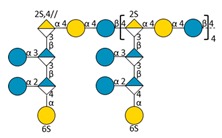	7491.37	10.27	1598.40	[M − 10H + 5IPR]^5−^
	**Supp. Species: E—(1** **⇨** **7 hexoses)**		7329.32 ⇨ 6357.00	10.29 ⇨ 10.43	1565.98 ⇨ 1371.51	[M − 10H + 5IPR]^5−^
	F	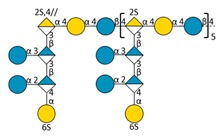	8989.64	10.77	1598.40	[M − 12H + 6IPR]^6−^
	**Supp. Species: F—(1** **⇨** **2 hexoses)**		8827.59 ⇨ 8665.54	10.79 ⇨ 10.80	1571.39 ⇨ 1544.36	[M − 12H + 6IPR]^6−^
